# Psychological Responses of Health Care Workers Are Strongly Associated With Pandemic Management

**DOI:** 10.3389/fpsyg.2022.902673

**Published:** 2022-07-07

**Authors:** Veronika Pacutova, Andrea Madarasova Geckova, Peter Kizek, Martin Novotny, Andrea F. de Winter, Sijmen A. Reijneveld

**Affiliations:** ^1^Department of Health Psychology and Research Methodology, Faculty of Medicine, P. J. Safarik University, Kosice, Slovakia; ^2^Department of Community and Occupational Medicine, University Medical Center Groningen, University of Groningen, Groningen, Netherlands; ^3^Institute of Applied Psychology, Faculty of Social and Economics Sciences, Comenius University Bratislava, Bratislava, Slovakia; ^4^I. Stomatology Clinic, University Hospital of Louis Pasteur, Faculty of Medicine, P. J. Safarik University, Kosice, Slovakia; ^5^Department of Infectious Disease and Travel Medicine, Faculty of Medicine, P. J. Safarik University, Kosice, Slovakia

**Keywords:** psychological responses, pandemic management, stress, COVID-19, health care workers, barriers and facilitative factors

## Abstract

**Background:**

Globally, governments adopted protective measures to stabilize the worsening COVID-19 pandemic situation. These measures had a large impact on health care workers (HCWs) and could make the work environment more stressful for them. Therefore, we explored the associations of the psychological responses of HCWs and pandemic management among Slovak health care workers during the second wave of the pandemic.

**Materials and Methods:**

We obtained data about psychological responses, COVID-19 experiences, information overload, non-adherence of the public, work stress and barriers and facilitators of health care provision from HCWs at the COVID-19-related departments of one hospital that mainly covered eastern Slovakia (215 health care professionals, 77.7% females, *M*/*SD* = 44.4). Data were analyzed using logistic regression models adjusted for gender.

**Results:**

Pandemic management was most strongly associated with psychological responses, such as irritability, sadness, anxiety, dizziness, and back pain, in health care workers. The non-adherence of the public had the strongest associated psychological impact on HCWs, mostly affecting anxiety and irritability (odds ratios, ORs varying from 3.9 to 7.0). From the barriers and facilitators of health care provision, the use of personal protective equipment had the most impact psychologically, whereas efficient department management was most protective, with ORs around 0.3.

**Conclusion:**

Pandemic management has a large impact on health care workers, and promoting resilience may seriously decrease job-related stress and associated psychological responses of HCWs.

## Introduction

The world continues to deal with the COVID-19 pandemic, which in Slovakia started in March 2020 and has unexpectedly continued in a series of waves. During the second wave, we noticed a serious rise in the number of infected patients, hospitalizations and even deaths, which forced the government to adopt protective measures to stabilize the pandemic situation regardless of the psychological and occupational impact on health care workers (HCWs), who were the most affected by the measures.

During the pandemic, health care workers have experienced a particularly stressful situation due to extreme workload, physical exhaustion, high risk of infection and social isolation, all of which have potentially severe psychological consequences (Du et al., 2020; Marinaci et al., 2020; Shoja et al., 2020; Slama et al., 2021). Protective measures led to the creating of COVID-related departments at hospitals, where many HCWs were redeployed and had to take on new competencies, learn to use new equipment and adjust to new work layouts. HCWs sometimes even found themselves there without proper specialization or training. They often worked overtime with the limited possibility to take breaks (Slama et al., 2021). The perception of personal risk was exacerbated by uncertainty, which was affected by the change in protective measures day by day (Chong et al., 2004). Due to the need to take protective measures, i.e., to wear personal protective equipment (PPE), regular drinking, eating and using restrooms became more difficult and were minimized, as was face-to-face communication, not to mention the consequences of the body overheating, skin irritation and the reduction of touching and visual sensations (Abiakam et al., 2021; Duan et al., 2021). Working staff were also afraid of understaffing and workload due to quarantine obligations or the sick leaves of their colleagues (Maunder et al., 2003). Studies show that between 59 and 96% of HCWs report moderate to severe stress (Du et al., 2020; Aly et al., 2021). It is unclear how long this pandemic will last, but, for example, with the SARS epidemic, stress among HCWs still persisted 1 year after the end of the epidemic (McAlonan et al., 2007).

Previous research has shown that the psychological responses of HCWs to pandemics occur in regard to anxiety, fear, panic attacks, posttraumatic stress, psychological distress, depressive tendencies, insomnia, tiredness, loneliness, frustration, anger, and grief (Maunder et al., 2003; Chong et al., 2004; Chua et al., 2004; Chew et al., 2020; Du et al., 2020; Huang et al., 2021). Moreover, somatic responses, such as headache, stomach pain, neck and back pain, also seem likely, given the connections between body and mind, as represented in the biopsychosocial model. Such responses have been previously shown in the health care environment (Nicholson et al., 2007; Yang et al., 2016; Onigbogi and Banerjee, 2019; Marinaci et al., 2020; Mohsin et al., 2021).

To our knowledge, evidence is lacking on the psychological responses of HCWs in Central Europe and the association of these responses with pandemic management strategies. Strong responses seem to be likely, however, as disease load and mortality were high, and pandemic management was mostly strict but also rapidly changing in these countries. We therefore aimed to assess whether various measures related to COVID-19 and COVID-19-management, such as COVID-19 experiences, information overload, non-adherence of the public, work stress and barriers and facilitators of health care provision, were related to the psychological responses of the HCWs concerned.

## Materials and Methods

### Sample and Procedure

We invited all practicing health care workers from the COVID-related departments (infection/anesthesiology and intensive care/pathology) of one hospital (covering Kosice region), one rescue service (covering Kosice region) and one dialysis services (covering all of Slovakia) through their employer, to participate in a cross-sectional survey administered during the second wave of COVID-19 pandemic, from May to September 2021 via an online or paper-based questionnaire. The questionnaire was specifically developed in cooperation with representatives of the participating HCWs and covered 11 areas (sociodemographic data, exposure to COVID-19, impacts on health care provision, adverse events, etc.), which were discussed as relevant. We arranged measurements based on their opinions, and the final version was piloted to assure clarity and suitability.

### Measures

**Psychological responses** of HCWs were measured by asking respondents how many times they suffered from headache, stomach ache, back pain, sadness, irritability, anxiety, insomnia, and dizziness in the last 6 months. Answers were dichotomized as: every day/more than once in a week vs. every week/month/rarely/never.

**Pandemic management** regarded a series of issues related to COVID-19 and the public management of this pandemic. First, this was the **COVID-19 experience** of HCWs, measured by asking respondents if they themselves had a serious experience with COVID-19, i.e., regarding hospitalization or death, personally, among close relatives, or within their work team (yes vs. no). **Information overload** regarded how frequently respondents followed pandemic news during the second wave of the COVID-19 outbreak from January till March 2021, and how much they were concerned about it. Combining these two questions, we divided the respondents into those who did not follow the news and were not concerned vs. those who followed the news several times per day and/or were highly concerned. Similarly, we asked HCWs how frequently they saw other people not following the pandemic measures during this period and how much they were concerned about the **non-adherence of the public**. Combining these two questions, we divided the respondents into those who almost always/always saw the non-adherence of the public and/or were highly concerned about them vs. never/sometimes saw the non-adherence of the public and were little/not at all concerned about them. **Work stress** was measured by asking HCWs if they were concerned about providing patient triage, applying work orders in handling patients, limitations due to emergency status and being delegated to perform their work without specialization. Responses were dichotomized as at least one vs. none. **Barriers and facilitators of health care provision** were factors that hindered and helped the HCWs, respectively, in providing health care during the second wave of the COVID-19 outbreak from January till March 2021. Potential barriers were (a) use of PPE, (b) lack of staff and (c) work exhaustion; these were dichotomized as partially/not limited vs. totally/significantly hindering for each factor. Potential facilitators were (a) efficient management at the department; (b) colleagues’ support and (c) public solidarity manifestation. Answers were dichotomized as highly vs. slightly/a little/not at all (Supplementary Appendix A).

### Statistical Analysis

First, we described psychological responses and the COVID-19 experience, information overload, non-adherence of the public, work stress, barriers, and facilitators of health care provision, which the Slovak HCWs reported as rates, means, and standard deviations (SDs). Second, we assessed the association of psychological responses with all COVID-19 pandemic management factors using logistic regression models adjusted for gender per each separate variable. We used IBM SPSS Statistics 23 for Windows for all analyses.

## Results

### Background Characteristics

We received 233 responses, which make up around 8% of the total number of employees, from which we later excluded those who did not report their gender (*n* = 6) and those who did not specify their profession (*n* = 12). The final sample included 215 respondents (77.7% females, mean age/*SD* = 44.4/ ± 10.2) and more than a half were nurses (52.1%). Most HCWs worked at the dialysis department (42.3%). HCWs complained mainly about back pain (36.9%). Around 50% had serious COVID-19 experiences, and about 60% had major concerns and/or frequently saw the non-adherence of the public. More than 30% of HCWs reported work exhaustion, lack of staff and use of PPE as the highest barriers in health care provision, while around 50% perceived department management and colleagues’ support as the most supportive facilitators (for more details, see Table 1).

**TABLE 1:** Demographic characteristics of the respondents (Slovakia 2021; *n* = 215 HCWs).

Characteristic	*N* (%)
**Gender**	
Women	167 (77.7)
Men	48 (22.3)
**Profession**	
Nurses	112 (52.1)
Doctors	69 (32.1)
Rescuers	27 (12.5)
Other HCWs	7 (3.3)
**Department of HCWs (dpt.)**	
*Hospital– local*	
Infection dept.	46 (21.4)
Anesthesiology and Intensive Care dept.	25 (11.6)
Pathology dept.	22 (10.2)
*Hospital– local and serving other hospitals*	
Dialysis services	92 (42.8)
Emergency service	30 (14.0)
**Psychological responses**	
Headache	52 (24.3)
Stomach ache	20 (9.5)
Back pain	79 (36.9)
Sadness	36 (16.8)
Irritability	46 (21.6)
Anxiety	34 (16.0)
Insomnia	37 (17.3)
Dizziness	19 (8.9)
**COVID-19 experience**	
Had serious COVID-19 experience (due to hospitalization or death)	108 (50.2)
**Information overload**	
Did not follow the news and not concerned	127 (59.3)
Either followed the news or highly concerned	65 (30.4)
Followed the news and highly concerned	22 (10.3)
**Non-adherence of the public**	
Never or sometimes saw non-adherence and not concerned	81 (37.9)
Either almost always/always saw non-adherence or highly concerned	75 (35.0)
Almost always/always saw non-adherence and highly concerned	58 (27.1)
**Work stress**	
At least 1 stressor (patient triage, work order, emergency status, no specialization)	84 (40.0)
**Barriers to health care provision**	
Use of PPE	72 (33.5)
Lack of staff	75 (35.0)
Work exhaustion	101 (47.4)
**Facilitators of health care provision**	
Efficient department management	107 (50.0)
Colleagues’ support	129 (60.3)
Public solidarity manifestation	50 (26.2)

### Associations of Psychological Responses With COVID-19 Experience, Information Overload, Non-adherence of the Public and Barriers and Facilitators of Health Care Provision

Pandemic management was most strongly associated with psychological responses, such as irritability, sadness, anxiety, dizziness, back pain, headache, stomach ache, and insomnia (in order, Table 2). Sadness and irritability were most frequently associated with psychological responses. Regarding the importance of measures of pandemic management, a serious COVID-19 experience did not have any significant association, whereas the non-adherence of the public, use of PPE and work stress had the most significant associations, with the strongest associations in the psychological area of anxiety and irritability (odds ratio/95% confidence interval, OR/CI: 7.0/2.13–22.3 and 7.0/2.59–18.8, respectively). Regarding barriers and facilitators of health care provision, the use of PPE had the strongest association (OR/CI: 4.6/2.15–9.81), whereas efficient department management was protective (OR/CI: 0.3/0.12–0.53). Moreover, public solidarity manifestation had an only association with HCWs’ insomnia (OR/CI: 4.2/1.21–14.6).

**TABLE 2:** Associations of psychological responses with COVID-19 experience, information overload, non-adherence of the public, barriers, and facilitators of health care provision.

	Headache OR (95%CI)	Stomach ache OR (95%CI)	Back pain OR (95%CI)	Sadness OR (95%CI)	Irritability OR (95%CI)	Anxiety OR (95%CI)	Insomnia OR (95%CI)	Dizziness OR (95%CI)
**COVID-19 experience**								
Serious experience	1.0 (0.51–1.79)	1.5 (0.59–3.90)	1.5 (0.86–2.69)	1.1 (0.51–2.18)	1.5 (0.76–2.89)	1.9 (0.88–4.1)	1.3 (0.65–2.73)	1.3 (0.51–3.45)
**Information overload**								
Followed or concerned Followed and concerned	1.2 (0.57–2.32) 1.2 (0.45–3.48)	2.2 (0.84–5.83) 0.6 (0.08–5.26)	1.5 (0.79–2.76) 1.4 (0.54–3.60)	**2.7 (1.18**–6.01)* **4.0 (1.38**–11.7)**	1.7 (0.82–3.42) 1.3 (0.43–3.88)	2.1 (0.95–4.81) 2.5 (0.77–7.88)	1.6 (0.74–3.53) 1.8 (0.58–5.35)	**3.1 (1.03**–9.17)* **4.4 (1.12**–17.0)*
**Non-adherence of the public**								
Non-adherence or concerned Non-adherence and concerned	**2.5 (1.10**–5.59)* **3.1 (1.33**–7.15)**	**7.6 (1.67**–36.0)** 4.5 (0.88–23.29)	1.1 (0.54–2.20) **2.9 (1.40**–5.95)**	**3.0 (1.06**–8.17)* **4.4 (1.59**–12.3)**	**3.9 (1.45**–10.5)** **7.0 (2.59**–18.8)***	**5.0 (1.57**–16.1)** **7.0 (2.13**–22.3)***	1.4 (0.55–3.35) **2.5 (1.01**–5.98)*	**5.4 (1.12**–25.9)* **5.4 (1.07**–26.8)*
**Work stress**								
At least one stressor	1.8 (0.98–3.56)	2.4 (0.94–6.19)	**1.9 (1.07**–3.47)*	**2.8 (1.32**–6.04)**	**3.4 (1.68**–6.73)***	**3.0 (1.35**–6.49)**	1.8 (0.89–3.80)	**5.4 (1.70**–17.3)**
**Barriers and facilitators of health care provision**								
Use of PPE	**2.5 (1.32**–4.76)**	2.2 (0.85–5.45)	**2.5 (1.37**–4.55)**	**4.6 (2.15**–9.81)***	**3.0 (1.54**–5.91)***	**2.3 (1.08**–4.85)*	1.1 (0.51–2.26)	**3.9 (1.45**–10.3)**
Lack of staff	**1.9 (1.01**–3.65)*	1.5 (0.60–3.90)	**2.0 (1.09**–3.56)*	**2.4 (1.13**–4.88)*	**2.2 (1.12**–4.23)*	2.0 (0.94–4.23)	2.0 (0.95–4.02)	1.7 (0.65–4.38)
Work exhaustion	1.5 (0.77–2.75)	1.6 (0.62–4.17)	**2.1 (1.19**–3.77)*	**2.9 (1.36**–6.36)**	**2.2 (1.12**–4.30)*	1.7 (0.80–3.59)	1.6 (0.75–3.25)	**4.7 (1.49**–14.6)**
Efficient dept. management	0.6 (0.33–1.21)	0.7 (0.28–1.82)	0.7 (0.42–1.33)	**0.3 (0.13**–0.62)**	**0.3 (0.12**–0.53)***	**0.3 (0.15**–0.75)**	0.6 (0.29–1.25)	0.5 (0.19–1.35)
Colleagues’ support	0.7 (0.38–1.34)	0.4 (0.16–1.04)	1.0 (0.54–1.72)	**0.5 (0.22**–0.96)*	**0.3 (0.17**–0.66)**	**0.4 (0.19**–0.84)*	0.6 (0.28–1.16)	0.7 (0.27–1.82)
Public solidarity manifestation	0.5 (0.21–1.15)	0.8 (0.26–2.77)	1.1 (0.55–2.16)	0.6 (0.23–1.54)	0.5 (0.21–1.26)	0.6 (0.24–1.71)	**0.2 (0.07**–0.82)*	0.2 (0.2–1.17)

***p < 0.001; **p < 0.01; *p < 0.05. Bold values are those which are significant.

## Discussion

We found that psychological responses of a somatic nature, such as headache, stomach ache and back pain, were most strongly associated with the non-adherence of the public and use of PPE, while the other psychological responses were strongly associated with more factors. Only one of the pandemic management factors, COVID-19 experience, was not associated with any psychological response. Regarding facilitators, department management, and colleagues’ support were most protective, while public solidarity manifestation was only associated with insomnia.

Psychological responses of a somatic nature, such as back pain, headache, and stomach ache, were associated with the non-adherence of the public and use of PPE (most strongly). They were also associated, less strongly, with a lack of staff, work exhaustion, and work stress. This confirms previous findings that somatic symptoms were highly associated with perceiving stress, especially in females, as well as with low job satisfaction, excessive workload, lack of staff, inadequate equipment/breaks, sleeping/eating problems or psychosocial stress (Hoogendoorn et al., 2002; Maleki et al., 2012; Tosunoz and Oztunc, 2017; Koyuncu and Karcioglu, 2018; Alnaami et al., 2019; Vinstrup et al., 2020). Moreover, headaches (new or worsening of pre-existing ones) develop demonstrably through the use of PPE for more than 4 h, when 61% admitted not removing masks until lunch break, which aligns with our results (Bharatendu et al., 2020; Hajjij et al., 2020; Ong et al., 2020; Contejean et al., 2021). Use of PPE may even cause “heat stress” (Moon et al., 2017; Lee et al., 2020). Perceiving stress from pandemic management can also affect contractions and movement of the gastrointestinal tract, and HCWs could recognize psychological responses more acutely (Harvard Health Publishing, 2021). HCWs from Saudi Arabia confirmed their feelings of nausea and stomach ache (62%) when thinking about the COVID-19 situation, as did American HCWs (56%) (Mental Health America, 2021; Mohsin et al., 2021). An explanation of our results might also be that HCWs were much more aware of the potential health consequences of COVID-19 disease on patients and were more likely to perceive stress when they saw that the public ignored measures that should basically protect them. They might be afraid of an increasing number of patients because of non-adherence and as a result could feel symptoms like headache, stomach ache and back pain. Headache and back pain were also associated with use of PPE, which might mean that they experienced prolonged use of PPE, dehydration, exhaustion, excessive sweating or a desire to find a comfort zone. We learned that feeling job-related stress from pandemic management was more likely symptomized by back pain and headache, with the non-adherence of the public and use of PPE being the strongest stressors, and we did not confirm any facilitators to be related to these kinds of psychological responses.

Psychological responses, such as irritability, sadness, anxiety, dizziness, and insomnia, were associated with the non-adherence of the public, use of PPE and work stress (most strongly). They were also associated, less strongly, with work exhaustion, information overload, and lack of staff. This is in line with other studies, which showed that sadness emerged in HCWs when the perceived information was insufficient, and levels of anxiety were higher in those working with COVID-19 patients or using PPE (bad-fitting, discomfort, many layers), whereas irritability was one of the main symptoms of vicarious traumatization (Alenazi et al., 2020; Li et al., 2020; Savitsky et al., 2020; García-Fernández et al., 2021; Ruskin et al., 2021). Furthermore, many HCWs reported that public solidarity manifestations, such as gifts or clapping hands, were nice, but some of them felt embarrassed (Rees et al., 2021). An explanation might be that HCWs perceived psychological job-related stress from bad-fitting or uncomfortable respirators, which increased the work of breathing or irritated their skin (Malik et al., 2020), as well as from frequency and adequacy of pandemic information. Relocating or joining a different team/department, new daily duties, operating new equipment, even without proper training, might also increase stress leading to the observed psychological responses (d’Ettorre et al., 2021; García-Fernández et al., 2021; Ruskin et al., 2021). Watching people not adhering to protective measures (wearing a face mask, using disinfection, keeping distances…), work stress and uncomfortable use of PPE might even cause traumatization to them (Li et al., 2020). Prioritizing patients in providing patient triage, applying work order, limitations due to emergency status and no specialization cause significant irritabilities for them. In contrast, HCWs might not be concerned so much about their own possibility of experiencing COVID-19 because of their better prognosis and shorter hospitalization (Diéz-Manglano et al., 2021; Yang et al., 2021). Public appreciation of their emotional and physical workload may have a protective effect only on their insomnia. We learned that perceiving job-related stress from pandemic management was more significant for these types of psychological responses, with the non-adherence of the public and use of PPE being the strongest stressors, whereas information overload was so in the first wave of the pandemic (Pacutova et al., 2021). Pandemic management made our HCWs more irritated, sad and anxious, whereas only efficient department management and colleagues’ support were sufficient to help them, and public solidarity manifestations were not enough.

### Strengths and Limitations

The main strength of this study is that we had a representative sample of HCWs from COVID-related departments during the second wave of pandemic despite their constant high work effort. Based on that, we were able to gather information about their psychological responses, COVID-19 experience, information overload, non-adherence of the public, work stress, barriers and facilitators of health care provision.

However, some limitations should be also considered. HCWs during the ongoing pandemic represent a hard to reach population. We recruited them via their employer, while their working locations were frequently changed, and we could not assure that the invitation would reach all of them. Moreover, not all of them might be willing to participate in any additional tasks due to exhaustion. Our sample size was relatively small resulting in not very robust results; however, we might hypothesize that those more burdened are those less reachable via a survey, so the results are more an underestimation than an overestimation of the real problems. Moreover, our study was cross-sectional, making it hard to establish causal relations between psychological responses and pandemic management. We did not do biological examinations including saliva or blood samples to assess hormonal and other biological indicators of stressful experiences.

### Implications for Practice

We found quite extensive psychological consequences of pandemic management, which implies that we should consider the impact of measures on HCWs, better account for their needs and strengthen psychological support. By increasing awareness that the non-adherence of the public significantly stresses our HCWs or by promoting better coping of HCWs with their negative feelings, we could assure fewer psychological responses related to this factor. Additionally regarding the use of PPE, we suggest that in regard to the challenges with breathing and prolonged use, short breaks during the day for drinking and eating, rest room visits to provide the comfort of breathing without a face mask or powered air purifying respirators for HCWs should be provided more often. Regarding potential dehydration, the consumption of sugary drinks and caffeine could be reduced and replaced by more moisture foods (fresh fruits, vegetables, yogurt, frozen food). Regarding skin irritation, PPE could be designed better and skin care simplified in various ways, such as the use of non-soap cleanser, mild fragrance-free moisturizers etc. (California Dental Association, 2020; Vidua et al., 2020).

We further found the strong association with work stress increased due to possible relocating, joining another team, new duties, new equipment and working without training/specialization, which implies that providing training and guidelines for HCWs on e.g., the proper use of PPE, patient triage and on duties after redeployment may help them fight job-related stress through better preparation (World Health Organization, 2020; U.S. Department of Veterans Affair, 2021). Our findings on the importance of department management and colleagues’ support imply that building peer support or a “COVID-19 Battle Buddy Support Programme,” sharing or celebrating successes and providing regular debriefings after shifts may help (Albott et al., 2020; Bielicki et al., 2020; National Guardian Program, 2021; Rieckert et al., 2021). In summary, pandemic management has a large impact on health care workers, and promoting resilience may seriously decrease job-related stress and associated psychological responses of HCWs.

## Data Availability Statement

The raw data supporting the conclusions of this article will be made available by the authors, without undue reservation.

## Ethics Statement

The study was appproved by the Ethics Committee of the Faculty of Medicine at P. J. Safarik University (14N/2020) and the Ethics Committee of Health Care Providers (2021/EK/05031; 813/2021). All data and information gathered from the documentation, including demographic and clinical data, were used in accordance with ethical standards as laid down in the 1964 Declaration of Helsinki and its later amendments or comparable ethical standards. Written informed consent to participate in this study was provided by the participants.

## Author Contributions

All authors listed have made a substantial, direct, and intellectual contribution to the work, and approved it for publication.

## Conflict of Interest

The authors declare that the research was conducted in the absence of any commercial or financial relationships that could be construed as a potential conflict of interest.

## Publisher’s Note

All claims expressed in this article are solely those of the authors and do not necessarily represent those of their affiliated organizations, or those of the publisher, the editors and the reviewers. Any product that may be evaluated in this article, or claim that may be made by its manufacturer, is not guaranteed or endorsed by the publisher.
