# Supplementary material for: Psychological Responses of Health Care Workers Are Strongly Associated With Pandemic Management
Source: Front Psychol. 2022 Jul 7;13:902673. doi: 10.3389/fpsyg.2022.902673 (PMC9306348; doi:10.3389/fpsyg.2022.902673)
Supplement: Supplementary file 1 [file Data_Sheet_1.pdf]

# Supplementary Appendix A

## Psychological responses

Q.1 In the last 6 months: How often did you have:

(a, every day; b, more than once a week; c, every week; d, every month; e, rarely/never)

- Headache
- Stomach ache
- Back pain
- Sadness
- Irritability
- Anxiety
- Insomnia
- Dizziness

## COVID-19 experience

Q.1 Did you have any serious experience of COVID-19 disease accompanied by hospitalisation or death?

- I had serious COVID-19 experience accompanied by hospitalisation or death
- I did not have such an experience
- Me/my family/my co-workers had a COVID-19 experience but without hospitalisation or death.

## Information overload

Q.1 Did you follow the pandemic news during the second wave (from January till March 2021)?

- Yes, several times per day
- Yes, at least once a day
- Yes, but not more than usual
- No

Q.2 Were you concerned about the pandemic news?

- No
- A little concerned
- Very concerned

## Non-adherence of the public

Q.1 How often did you meet a patient or another person who did not follow the pandemic measures (e.g. did not wear face masks) from January till March 2021?

- Never
- Sometimes
- Almost always
- Always

Q.2 Were you concerned about the non-adherence of the public (not wearing face masks)?

- No

- A little concerned
- Very concerned

**Work stress**

Q.1 Were you ever concerned about:

(a, yes; b, no)

- Providing patient triage
- Applying a work order
- Limitations due to emergency status (no possibility to take vacations or resign)
- Performing work duties without specialisation

**Barriers of health care provision:**

Q.1 How much did the following circumstances hinder you in providing health care?

(a, limited; b, significantly limited; c, partially limited; d, not limited)

- Use of PPE
- Lack of staff
- Work exhaustion in the team

**Facilitators of health care provision:**

Q.1 How much did the following circumstances help you in providing health care

(a, highly; b, slightly; c, a little; d, not at all)

- Efficient department management
- Colleagues' support
- Public solidarity manifestation
